# Genetic Profiles Affect the Biological Effects of Serine on Gastric Cancer Cells

**DOI:** 10.3389/fphar.2020.01183

**Published:** 2020-07-31

**Authors:** Jun Li, Hongzhang Xue, Zhen Xiang, Shuzheng Song, Ranlin Yan, Jun Ji, Zhenggang Zhu, Chaochun Wei, Yingyan Yu

**Affiliations:** ^1^ Department of Surgery of Ruijin Hospital, Shanghai Institute of Digestive Surgery, and Shanghai Key Laboratory for Gastric Neoplasms, Shanghai Jiao Tong University School of Medicine, Shanghai, China; ^2^ Department of Bioinformatics and Biostatistics, School of Life Sciences and Biotechnology, Shanghai Jiao Tong University, Shanghai, China; ^3^ SJTU-Yale Joint Center for Biostatistics and Data Science, Shanghai Jiao Tong University, Shanghai, China

**Keywords:** gastric cancer, MGC803, HGC27, microenvironment, serine

## Abstract

A high serine content in body fluid was identified in a portion of patients with gastric cancer, but its biological significance was not clear. Here, we investigated the biological effect of serine on gastric cancer cells. Serine was added into the culture medium of MGC803 and HGC27 cancer cells, and its influence on multiple biological functions, such as cell growth, migration and invasion, and drug resistance was analyzed. We examined the global transcriptomic profiles in these cultured cells with high serine content. Both MGC803 and HGC27 cell lines were originated from male patients, however, their basal gene expression patterns were very different. The finding of cell differentiation-associated genes, ALPI, KRT18, TM4SF1, KRT81, A2M, MT1E, MUC16, BASP1, TUSC3, and PRSS21 in MGC803 cells suggested that this cell line was more poorly differentiated, compared to HGC27 cell line. When the serine concentration was increased to 150mg/ml in medium, the response of these two gastric cancer cell lines was different, particularly on cell growth, cell migration, and invasion and 5-FU resistance. In animal experiment, administration of high concentration of serine promoted cancer cell metastasis to local lymph node. Taken together, we characterized the basal gene expressing profiles of MGC803 and HGC27. The HGC27 cells were more differentiated than MGC803 cells. MGC803 cells were more sensitive to the change of serine content. Our results suggested that the responsiveness of cancer cells to microenvironmental change is associated with their genetic background.

## Introduction

Gastric cancer (GC), which ranks the fifth in incidence and the third in mortality worldwide, is one of the most common malignant tumors of digestive tract. This malignancy commonly occurs in Asian countries, especially in China, Japan, and South Korea (Torre et al., 2015; Bray et al., 2018). Because there is no sensitive and specific diagnostic method for GC, most patients have developed to advanced stage and lost the best time for surgical treatment when they were diagnosed. Therefore, novel detection methods and molecular biomarkers for early diagnosis have been extensively explored (Dou et al., 2018; Song et al., 2019).

Our team has been working on translational research and exploring the pathogenesis of gastric cancer. In a previous study on urine metabolites, a group of amino acids were found to be abnormally elevated in GC patients than that in healthy controls. Those amino acids include alanine, glycine, valine, serine, isoleucine, threonine, proline, methionine, tyrosine, and tryptophan. Among them, all the values of the area under curve (AUC) of the diagnostic curves of threonine, serine, and alanine were above 0.8, indicating the good diagnostic value for GC. Moreover, some of the amino acids showed predictive potential for prognosis (Chen et al., 2016). These findings implied that increased certain amino acids in body fluid might have biological significance on GC development.

Tumor is the overgrowth of cell clusters and the result of abnormal metabolism. Compared with normal cells, tumor cells demand more nutrients for their rapid proliferation and metastasis, and reshape a variety of anabolic and catabolic pathways in the nutrient-deficient microenvironment (Davidson et al., 2016; Bubnovskaya and Osinsky, 2020). Amino acids are important metabolites of tumor cells. Elevation of some amino acids in microenvironment could provide favorable conditions for tumor growth, metastasis, drug resistance, and others. For example, Saito et al. reported that elevated leucine in microenvironment can promote cell proliferation in breast cancer, and induce resistance to tamoxifen (Saito et al., 2019). Engel et al. found that serine content of microenvironment determined the growth and survival of glioblastoma (Engel et al., 2020). In lung cancer, the imbalance of kynurenine to tryptophan ratio in microenvironment was closely related to the resistance for immune checkpoint inhibitor (Li et al., 2019). In this study, we explored the influence of high serine concentration on multiple biological behaviors, as well as the possible molecular mechanisms for GC.

## Materials and Methods

### Cell Culture and Reagents

GC cell lines MGC803 and HGC27 were stored at the Shanghai Institute of Digestive Surgery. Both cell lines were cultured in RPMI-1640 medium (Gibco, USA) supplemented with 10% fetal bovine serum (Gibco, USA) and 5 μg/ml penicillin-streptomycin in a humidified incubator at 37°C with 5% CO2. In addition, RPMI-1640 medium supplemented with 1% fetal bovine serum was prepared for experiments and stored at 4°C.

Serine (Purity>98.5%, LR, BBI Life Sciences, China) was fully dissolved with phosphate buffer (1 x PBS) to the concentration of 150 mg/ml and was filtered with 0.22 µm aperture needle filter (Millipore, United States) and then stored at 4°C. Puromycin (Cat. #ANT-PR-5B, Invivogen, USA), rabbit GFP monoclonal antibody (Proteintech, 50430-2-AP, China), HRP-labeled goat anti-rabbit antibody (Servicebio, GB23305, China), 5-Fluorouracil (5-FU, Shanghai Xudong Haipu Pharmaceutical Co., Ltd., China) and Cryptotanshinone (2.5 μM, S2285, SELLECK, Houston, USA) were stored at −20°C.

### Determining the Serine Concentration

In order to find proper concentration of serine in functional experiments, we designed five serine concentrations of 0, 30, 150, 300, and 600 μg/ml. Then we calculated migration cells through transwell experiment. Briefly, MGC803 or HGC27 cells (8×10^4^/well) were added onto a 24-well plate (Corning Life Science, Acton, MA, USA). A 700-μl RPMI-1640 medium complemented with 10% fetal bovine serum (FBS), and different concentration of serine was added to the lower chamber of the 24-well plate, and 300 μl FBS-free medium to the upper chamber. After incubation for 24 h, we stained the GC cells on the inserts by 0.5% crystal violet for 20 min at room temperature. The upper remaining cells of the inserts were removed with cotton swabs. Finally, the migrated GC cells were counted at the 200× under the Olympus BX50 microscope (Olympus Optical Co. Ltd., Tokyo, Japan), and photographed by Nikon Digital Sight DS-U2 (Nikon, Tokyo, Japan) camera. Five visual fields were randomly chosen to calculate the number of migrated cells.

### RNA Sequencing and Data Analysis

HGC27 and MGC803 cells (1×10^6^) were planted in 10cm dish with RPMI-1640 medium and 10% FBS. The next day, the cells were divided into two groups. One is in standard RPMI-1640 medium plus with serine 30 μg/ml and 1% FBS. The other is in standard RPMI-1640 medium with 1% FBS and an additional serine of 150 μg/ml. Three repeated wells were set for each group. After incubation for 48h, cells were collected into 1.5ml tube with 1 ml Trizol (Invitrogen, USA) for RNA extraction. RNA purification was checked by NanoPhotometer^®^ spectrophotometer (IMPLEN, CA, USA), and RNA integrity was evaluated by Bioanalyzer 2100 system (Agilents, CA, USA). After the RNA quality control, sequencing libraries were generated using NEBNext^®^ UltraTM RNA Library Kit for Illumina^®^ (NEB, USA). RNA sequencing was performed on Illumina Novaseq6000 platform (Jiayin Biomedical Technology Co., Ltd., Shanghai, China). Indexes clustering were performed on cBot Cluster System using TruSeq PE Cluster Kit v3-cBot-HS (Illumia). HTSeq v0.6.0 was used to count the reads numbers mapped to each gene, and then the FPKM (Fragments Per Kilobase per Millions base pairs) of each gene was calculated based on the length of each gene and reads counts. The DESeq2 algorithm was used to filter the expression of differential genes. Differential genes between MGC803 and HGC27 cell lines were plotted with R software (version, R i386.3.6.3). The differential genes (at the standard |log2 (fold change)| >0.585 and P<0.001) between high-serine medium group and standard medium group were analyzed. The RNA-seq data of cancer cell lines treated by serine could be found in SRA database (PRJNA638214).

### Cell Proliferation Assay

In the CCK8 assays, MGC803 or HGC27 cells (2 × 10^3^/well) were added into the 96-well plate with six repeated wells for each condition under RPMI-1640 medium 100 μl with 10% FBS incubation for 24 h. Then, the medium was replaced by RPMI-1640 medium with 1% FBS. The experimental groups were divided as above. On the first, second, and third days, each well was added 100 μl 10% CCK8 solution diluted in medium with 1% FBS for 2 h incubation. The OD values at 450nm wavelength were measured by a spectrophotometry (BioTek, Vermont, USA).

### Colony Formation Assay

In the colony formation assay, cancer cells (1 × 10^3^/well) were added to six-well plate, and incubated with 2.5 ml mediums with 1% FBS for 3 days. And then, the medium was replaced with 2.5 ml above medium for another four days. The chemical cryptotanshinone was added at the 7th day. On the 14th day, the cell colony were fixed and dyed with 0.5% crystalline purple diluted in methanol for 20 min. After washing with clean water, the colony was photographed and counted by observing visible colony units in five fields.

### Cell Migration and Invasion Assays

Cell migration and invasion assay were performed by using transwell chambers (Corning, Lowell, MA, USA) coated with or without matrigel (BD Biosciences, Bedford, MA). MGC803 or HGC27 cells (8× 10^4^/well) were added to the upper chamber and cultured for 48 h at 37°C with 5% CO2. RPMI-1640 medium 700 μl with 10% FBS and different serine concentration was added to lower chambers and 200 μl medium without FBS but containing different serine concentration or cryptotanshinone was added to upper chamber. After incubation for 48 h, cells on the inserts were fixed and stained with 0.5% crystal violet diluted by methanol for 20 min at room temperature. The upper remaining cells of the inserts were removed with cotton swabs. Permeating cells were counted under the inverted microscope in five random fields. The photographs were taken under the 200× field (Nikon, Tokyo, Japan).

### 5-FU Sensitivity Assay

MGC803 or HGC27 cells (5 × 10^3^/well) were added into 96-well plates, and incubated in 100 μl high serine medium (150 μg/ml) or standard medium with 1% FBS for 24 h. Then, the medium was replaced with above two mediums containing gradient concentrations of 5-FU (0, 0.25, 0.5, 1, 2, 4, and 8 μg/ml). After incubation for 48 h, 100-μl solution of 10% CCK8 was added for 2 h, and OD values at 450nm were measured by a spectrophotometry (BioTek, Vermont, USA).

### Popliteal Lymph Node Metastasis

A total of twelve 5-week-old female nude mice (BALB/C nude, Beijing Vitolihua Experimental Animal Technology Co., Ltd.) were randomly divided into two groups. One is high serine group, and another is standard group (Normal). The mice were raised at the Shanghai Experimental Animal Research Center. The experiment was approved by the Research Ethics Committee of Shanghai Jiaotong University School of Medicine. A total of 5 × 10^6^ HGC27 cells were injected into the left rear foot pad. The detailed steps were reported in the previous study (Xiang et al., 2019). The experimental animals were injected intraperitoneally with serine solution (2g/kg) (Sasaki et al., 2017), and control animals were injected intraperitoneally with 150-μl PBS solution. The first three injections were given every two days, then changed to every three days. Four weeks later, the mice were sacrificed, and primary tumors in foot pad and popliteal lymph nodes were removed for analysis. The size of the popliteal lymph nodes were calculated using the formula (volume=length ×width ×width/2). The popliteal lymph nodes were fixed with 4% formalin and slices were made for examination.

All removed lymph nodes were fixed by formalin and embedded in paraffin. The 4-μm thick slices were made to perform H&E and IHC staining by streptavidin-peroxidase method. Rabbit anti-GFP monoclonal antibody (1:100, Proteintech, 50430-2-AP, China) and HRP-labeled goat anti-rabbit antibody (1: 200, Servicebio, GB23305, China) were used. After staining, semi-quantitative analysis of GFP was conducted according to the proportion and intensity of stained tumor cells. The photographs were taken under the Nikon Digital Sight DS-U2 (Nikon, Tokyo, Japan) at low- and high-power fields.

### Statistical Analysis

All data were performed using GraphPad Prism 8.0 (Inc., La Jolla, CA, USA). The inter-group differences were analyzed by the Student’s t test. The positive popliteal lymph node metastasis was analyzed by χ2 test. P<0.05 was considered statistically significant.

## Results

### The Effect of Serine on Cancer Cell Migration

**Figure 1:**
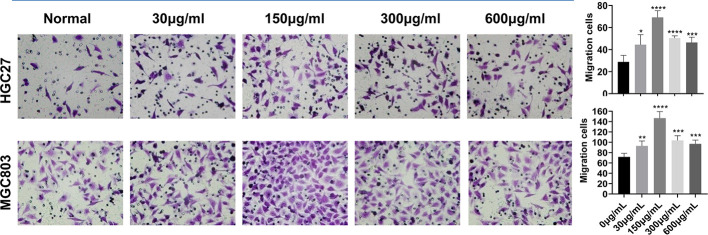
The effect of different serine concentration on cancer cell migration. Five serine concentrations from 0 to 600 μg/ml in culture medium were set. After incubating MGC803 or HGC27 cells in mediums with different serine concentrations for 24 h, the optimum serine concentration of 150 μg/ml medium was observed in both cancer cell lines. In addition, the migratory ability of MGC803 cells was higher than that in HGC27cells at high serine condition. These data are representative examples taken from one of the three experiments. “*” represents P < 0.05, comparing to “Normal” group, **P < 0.01, ***P < 0.001, ****P < 0.0001.

Five concentrations of serine (0, 30, 150, 300, and 600 μg/ml) were added to RPMI-1640 medium. After incubating MGC803 or HGC27 cells with the above five mediums for 24 h, the medium with 150 μg/ml serine showed the highest effect in promoting cell migration (69.2 ± 5.4) compared to that in RPMI-1640 medium (28.8 ± 5.3) in HGC27 cells (P<0.0001). The similar trend was observed in MGC803 cells (146.6 ± 11.5 vs 71.6 ± 6.2, P<0.0001) (**Figure 1**). In addition, the MGC803 cells showed more migrating cells than that of HGC27 cells in high serine culture medium (146.6 ± 11.5 vs. 69.2 ± 5.4, P<0.0001).

### The Effect of Serine on Global Transcriptomic Profiles on Gastric Cancer Cells

**Figure 2:**
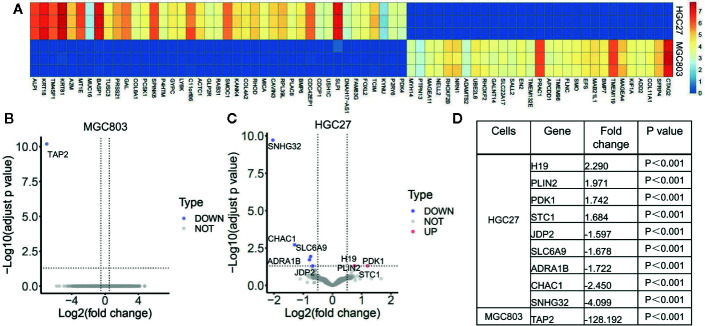
The effect of serine on global transcriptomic profiles of MGC803 and HGC27 cells. **(A)** Image of hierarchical clustering analysis for basal expression profiles of MGC803 and HGC27 cancer cells. The heatmap is presented by the FPKM of each differential gene. Row represents experimental cells, and column represents genes. The up-regulated genes are marked by light red color, and down-regulated genes are marked by dark blue color. **(B, C)** The volcano plots of differential gene expression of MGC803 cells and HGC27 cells in high serine group and standard medium group. **(D)** The fold change of differential genes in MGC803 and HGC27 cells. These data are representative examples taken from one of the three experiments.

Because 150 μg/ml serine promoted cancer cell migration, we analyzed the effect of serine on global transcriptomic profiles of these two cancer cells. By RNA-Seq analysis, although the basal expression of Y chromosome-located genes of MGC803 and HGC27 cells were highly associated (coefficient R=0.794), the expression of thousands of autosomal genes were very different. For instance, the top ten highly expressed genes in HGC27 cells were ALPI, KRT18, TM4SF1, KRT81, A2M, MT1E, MUC16, BASP1, TUSC3 and PRSS21. The expression levels of each of those genes in HGC27 cells were over ten thousand-fold higher than that in MGC803 cells (**Figure 2A**). We separately analyzed the effect of high serine on MGC803 cells and HGC27 cells. Under the strict condition, only one gene TAP2 was differentially expressed between high-serein group and standard medium group in MGC803 cells (**Figure 2B**), while nine genes were differentially expressed in HGC27 cells. Among those different genes, four were up-regulated, and five were down-regulated (**Figure 2C**). The fold changes of different genes in both cell lines were presented in **Figure 2D**. Notably, gene PDK1, which involves in the JAK/STAT3 molecular pathway and associates with cell growth and invasion, was one of the differentially increased genes in the high serine group.

### The Effect of Serine on Cell Growth

**Figure 3:**
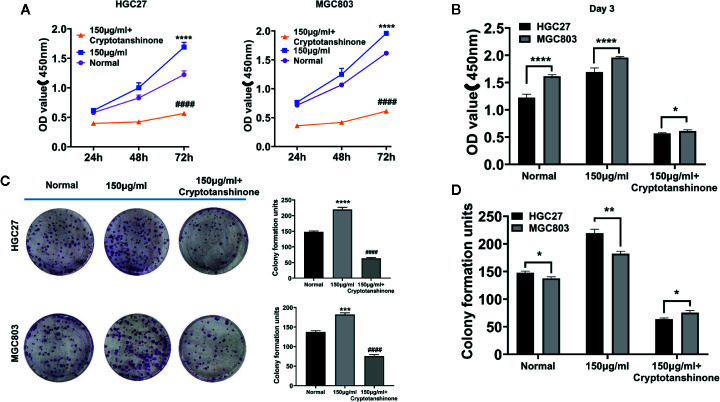
The proliferation-enhancing effect of high serine on HGC27 and MGC803 cells. **(A)** CCK8 assay on the proliferation-enhancing effect of high serine on HGC27 and MGC803 cells. **(B)** CCK8 assay on the cell growth of HGC27 and MGC803 cells. **(C)** Colony formation assay on the proliferation-enhancing effect on HGC27 and MGC803 cells. **(D)** The comparison of different effects on colony formation between HGC27 and MGC803 cells. These data are representative examples taken from one of the three experiments. “*” represents P < 0.05, comparing to “Normal” group, **P < 0.01, ***P < 0.001, ****P < 0.0001. "^####^" represent P < 0.0001, compared to high-serine group.

CCK-8 assay showed that the cell proliferation ability of HGC27 and MGC803 cells were significantly increased in high serine group (1.69 ± 0.07, 72 h and 1.96 ± 0.01, 72 h, respectively), compared with that in standard medium (1.22 ± 0.05, 72 h, and 1.62 ± 0.05, 72 h, respectively, p<0.0001). The cell proliferative ability could be suppressed by cryptotanshinone (0.52 ± 0.01, 72 h, P<0.0001) in high-serine condition (0.57 ± 0.01, 72 h, 0.55 ± 0.01, 72 h, respectively, P < 0.0001, **Figure 3A**). The cell proliferation promoting effect of high serine in MGC803 cells was stronger than that in HGC27 cells (P<0.001, **Figure 3B**). In addition, we observed much more colony formation of HGC27 cells in high serine group (219.3 ± 5.7), compared with that in standard medium (147.7 ± 2.5, p<0.001). The cryptotanshinone could suppress colony formation in high serine condition (47.3 ± 3.7, P<0.001, **Figure 3C**). In MGC803 cells, colony formation was higher in high serine group (182.3 ± 3.7), compared with that in standard medium (137.3 ± 2.5, p<0.01). The cryptotanshinone suppressed colony formation in high-serine-condition (51.7 ± 2.5, P<0.001). There are more colony formation in HGC27 cells that in MGC803 cells with high serine concentration (P<0.01, **Figure 3D**).

### The Effect of Serine on Cell Migration and Invasion

**Figure 4:**
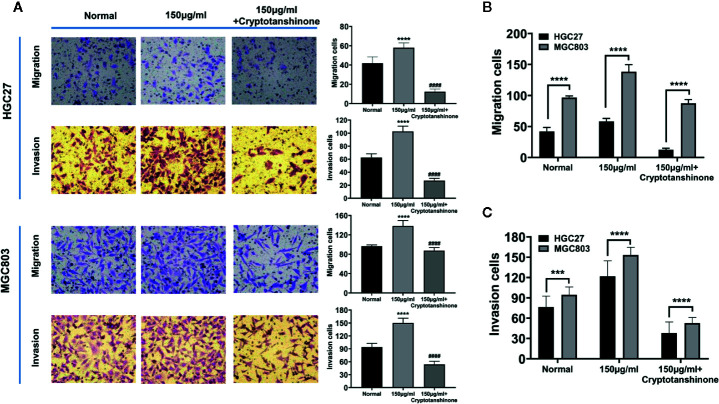
Effects of high serine condition on cell migration and invasion of MGC803 and HGC27 cells. **(A)** The enhancing effect on cell migration and invasion under high serine condition. High serine in medium significantly promotes migration and invasion of HGC27 and MGC803 cells. Incubation with chemical cryptotanshinone suppresses cell migration and invasion. **(B)** Comparison of the difference on cell migration between HGC27 and MGC803 cells under the same cell culture conditions. **(C)** Comparison of the difference on cell invasion between HGC27 and MGC803 cells under the same cell culture conditions. These data are representative examples taken from one of the three experiments. "***" and "****" represent P < 0.001, and P < 0.0001, compared to normal group. "^####^" represent P < 0.0001, compared to high-serine group.

High serine promoted cell migration of HGC27 cells (58.0 ± 4.4), compared to standard medium (41.8 ± 5.9 cells, P<0.0001), The cryptotanshinone significantly suppressed cell migration in high-serine condition (5.2 ± 1.2, P<0.0001). Similar experimental results were obtained in MGC803 cancer cells (**Figure 4A**). In addition, the migration cells of MGC803 in high serine group were much more than that in HGC27 cells (138.2 ± 8.5 vs. 58.0 ± 4.4, P < 0.0001, **Figure 4B**). In cell invasion assay, the strongest invasion ability of HGC27 cells (102.6 ± 7.3) was observed in high serine group, compared with that in standard medium (62.4 ± 5.2 cells, P < 0.0001). The cryptotanshinone significantly suppressed cell invasion of HGC27 cells in high-serine condition (14 ± 1.7, P < 0.0001). Similar experimental results were obtained in MGC803 cells (**Figure 4A**). We noticed that the cell invasion ability in high serine group in MGC803 were stronger than that of HGC27 cells (149.8.2 ± 10.0 vs. 102.6 ± 7.3, P<0.0001, **Figure 4C**).

### The Effect of Serine on 5-FU Sensitivity

**Figure 5:**
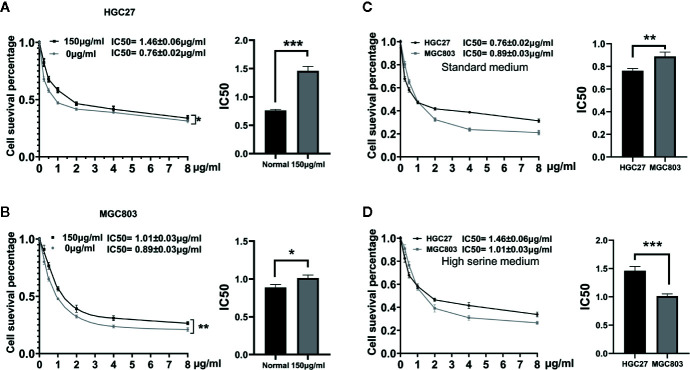
Drugs sensitivity analyzed by CCK8 assay. **(A, B)** Dose-response curves and drug concentration of IC 50 of 5-FU between high serine group and standard medium in HGC27 and MGC803 cells. The sensitivity to 5-FU is reduced in high serine condition. **(C)** The difference of IC50 between HGC27 and MGC803 cells in standard medium. **(D)** The difference of IC50 between HGC27 and MGC803 in high serine medium. These data are representative examples taken from one of the three experiments. “*” represents P < 0.05, comparing to “Normal” group, **P < 0.01, ***P < 0.001,

To evaluate the effect of high serine on chemosensitivity, MGC 803 and HGC27 cells were treated with 5-FU. The IC50 of 5-FU on HGC27 cells was higher in high serine group (1.46 ± 0.06 μg/ml), compared to standard medium group (0.76 ± 0.02 μg/ml, p<0.01) (**Figure 5A**). Similarly, the IC50 of 5-FU on MGC803 cells was higher in high serine group (1.01 ± 0.03 μg/ml) than that in standard medium group (0.89 ± 0.03 μg/ml, p<0.05) (**Figure 5B**). In standard medium, the IC50 of 5-FU in MGC803 cells (0.89 ± 0.03 μg/ml) was higher than that in HGC27 cells (0.76 ± 0.02 μg/ml, P<0.05, **Figure 5C**). However, in high serine condition, the IC50 of 5-FU in MGC803 cells (1.01 ± 0.03 μg/ml) was lower than that in HGC27 cells (1.46 ± 0.06 μg/ml, P<0.01) (**Figure 5D**). It means that the sensitivity to 5-FU on MGC803 cells was 1.45-fold higher than that in HGC27 cells in high-serine condition.

### The Effect of Serine on Lymph Node Metastasis In Vivo

**Figure 6:**
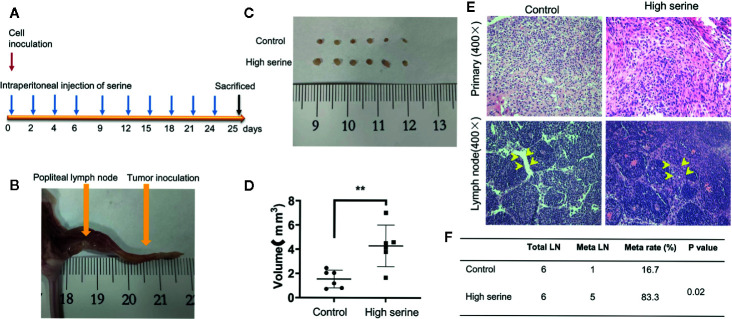
Cancer metastasis analysis in nude mice. **(A)** The schematic of time points for cancer inoculation and serine administration. **(B)** Examination of foot pad and popliteal lymph nodes in nude mice. **(C)** The size differences of popliteal lymph nodes between experimental group and control group. **(D)** The volume differences of popliteal lymph nodes measured between experimental group and control group. **(E)** The primary tumor (up) and nodal metastases (down) were examined by H&E staining (400X). Compared to control, more metastatic cancer cells were observed in lymphatic sinuses (arrows). **(F)** The metastatic rate of popliteal lymph nodes was significantly different between the two groups. **P < 0.01.

To examine the possible effect of serine on promoting cancer cell metastasis *in vivo*, we established a popliteal lymph node metastatic model in nude mice. In experimental group, high concentration of serine (2g/kg) was administrated *via* abdominal cavity, while PBS was administrated *via* abdominal cavity as control (**Figure 6A**). After ten times administration of serine, the mice were sacrificed, and the popliteal lymph nodes were examined (**Figure 6B**). It was found that the sizes of the popliteal lymph nodes in high serine group were significantly larger than that in controls (4.29 ± 1.56 mm^3^ vs.1.54 ± 0.67 mm^3^, P<0.01, **Figures 6C, D**). We further detected the metastatic cancer cells histologically, and found that the metastatic rate of cancer cells in lymph nodes of high serine group was significantly higher than that in controls (83.3% vs 16.7%, P<0.05, **Figures 6E, F**).

## Discussion

Gastric cancer is the most common primary malignancy of digestive tract with a dismal prognosis. Multimodal therapeutic approaches have been used in gastric cancer treatment, such as surgical resection, chemotherapy, and radiotherapy. However, the response to therapies is very different, which could be attributed to different genetic background of individuals. Cancer cells are frequently used as experimental model in basic and clinical studies. MGC803 and HGC27 cell lines have been used for many years in gastric cancer research, however, the difference of their genetic background have not been reported (Tamura et al., 1996; Zhang et al., 2004; Jin et al., 2015). By global transcriptomics profiling for both cell lines, we characterized the differential expression of genes between these two cell lines. Among those, HGC27 cell line showed much more expression of differentiated genes compared to MGC803 cells. MGC803 cells had more genes of poorly differentiated cells. Cancer cells with different genetic backgrounds may response differently to external stimulation, including drug treatment. There were reports indicated that microenvironmental serine may alter cancer proliferation and invasion. For instance, Engel and colleagues reported that tumor microenvironment with high serine concentration contributed to rapid cell growth in glioblastoma. Interfering serine metabolism could be a plausible therapeutic target (Engel et al., 2020). It was reported that the proliferative ability of cancer cells could be enhanced by high content of microenvironmental serine (Labuschagne et al., 2014). Serine promoted cancer growth in prostate cancer (Reina-Campos et al., 2019). Up to date, there is no report on the biological effects of microenvironmental serine on gastric cancer.

In our previous study, significantly increased amino acids, including serine, were found in urine of patients with gastric cancer, implying a disturbed metabolism of amino acids (Chen et al., 2016). Amino acids serve the essential functions of redox balance, energetic regulation, biosynthesis, and homeostatic maintainence for living things (Lieu et al., 2020; Vettore et al., 2020). Serine is one of the pivotal nutrients for cell growth in routine RPMI-1640 culture medium with 30 μg/ml concentration (Mossinger, 1991). In the current study, in order to find out a proper high serine environment, we screened several concentrations and found that 150 μg/ml serine is the optimal concentration on enhancing cell growth and invasiveness of cancer cells. Our study showed that MGC803 cells and HGC27 cells had different biological behaviors no matter in standard condition or in high serine condition. This phenomenon could be explained by their different genetic backgrounds. These two cell lines were established from male patients, because they showed similar expression of genes of Y chromosome. However, the top ten significantly decreased genes (ALPI, KRT18, TM4SF1, KRT81, A2M, MT1E, MUC16, BASP1, TUSC3 and PRSS21) in MGC803 cell were over ten thousand-fold lower than that in HGC27 cells. Most of those genes were closely related to epithelium differentiation (Wang et al., 2008; Green et al., 2009; Shin et al., 2014; Kratochvilova et al., 2015; Wang et al., 2016; Conway et al., 2019; Golob-Schwarzl et al., 2019). This result indicated that the MGC803 cell originated from poorly differentiated gastric cancer, while HGC27 cell might come from relatively differentiated gastric cancer. Our results will provide important reference for selecting proper cell models for future research. The higher proliferating and invasive ability of MGC-803 cells may attribute to its lower differentiation.

We treated MGC803 and HGC27 cells with same concentration of serine *in vitro*. The change of gene expression pattern was very different. Upon analysis, only TAP2 (transporter 2, an ATP binding cassette subfamily B member) gene showed significant down-regulation in high serine condition in MGC803 cells. TAP2 encodes a membrane-associated protein, which is a member of the MDR/TAP subfamily. Members of the MDR/TAP subfamily are involved in multidrug resistance (Lage et al., 2001). Since high serine stimulation significantly reduced TAP2 expression, the MGC803 cells showed increased sensitivity to 5-FU treatment after up-regulation of microenvironmental serine content. However, the molecular mechanisms should be explored further. In HGC27 cells, high serine condition resulted in different gene expression pattern. The increased expression of genes included oncogenic gene PDK1. PDK1 encodes a pyruvate dehydrogenase kinase 1, which is one of the major enzymes responsible for the regulation of homeostasis of carbohydrate fuels in mammals (Ba et al., 2020). The aberrant activation of PDK1 and its downstream effectors has been reported to involving in pathological phenotypes such as uncontrolled cell proliferation, apoptosis escape, invasion, and metastasis (Qian et al., 2020a), which may explain why HGC-27 cells showed higher metastatic ability in high serine administration in animal experiment. Some researches showed that PDK1 plays a role in chemoresistance in different types of malignancies, and targeting PDK1 could be a selection for promoting chemosensitization (Emmanouilidi and Falasca, 2017; Qian et al., 2020b). Recently, Yuan and colleagues reported that cryptotanshinone, originally a STAT3 inhibitor showed multiple suppressing activity on eight targets with anticancer potential, including MAP2K1, RARalpha, RXRalpha, PDK1, CHK1, AR, Ang-1 R, and Kif11 (Yuan et al., 2014). Those targets are related to promoting cancer growth. Their results provided a clue for the study of the anticancer effects and mechanisms of cryptotanshinone (Gagliardi et al., 2018). In our study, cryptotanshinone showed the suppressing effect on both cancer cell lines. It implied that microenvironmental high serine may activate multiple pathways. The biological behaviors observed in our study suggested that cancer cells might respond differently to microenvironmental serine and the difference might be related to different genetic background and original histological classification. Carter and coworkers reported that sensitivity of radiotherapy of colorectal cancer depended on the genetic background of cancers (Carter et al., 2019).

## Conclusions

This study demonstrated the global basal expression profiles of MGC803 and HGC27 cells. We found HGC27 cell was more differentiated than MGC803 cell. Although high serine condition could enhance the malignant behaviors in both cancer cells, MGC803 cells are more sensitive to change of serine concentration. This discrepancy could be attributed to different genetic background of cells. Since MGC803 and HGC27 cell lines are commonly used in basic experiments of gastric cancer. This finding will provide important references for cell selection for future research. Moreover, microenvironmental serine content could affect multiple biological behaviors, such as cell growth, cell migration, and invasion, and chemoresistance. Altering serine content of tumor microenvironment could be a new direction for cancer therapy in future.

## Data Availability Statement

The sequencing data has been deposited into BioProject (https://www.ncbi.nlm.nih.gov/sra/?term=PRJNA638214).

## Ethics Statement

The animal study was reviewed and approved by Shanghai Jiao Tong University School of Medicine.

## Author Contributions

YY and JL designed the study. JL, ZX, SS, RY, and JJ performed the experiments. HX and CW processed sequencing data and analyzed data. JL and YY wrote the manuscript. All authors contributed to the article and approved the submitted version.

## Funding

This project was partially supported by the Chinese National Key Program (2016YFC1303200 and MOST-2017YFC0908300), the National Natural Science Foundation of China (81772505), Shanghai Science and Technology Committee (18411953100), the cross-institute innovation foundation of Shanghai Jiao Tong University (YG2017ZD01), the Innovation Foundation of Translational Medicine of Shanghai Jiao Tong University School of Medicine (15ZH4001, TM201617 and TM 201702), and Technology Transfer Project of Science & Technology Dept. Shanghai Jiao Tong University School of Medicine. The funders had no role in study design, data collection and analysis, decision to publish, or preparation of the manuscript.

## Conflict of Interest

The authors declare that the research was conducted in the absence of any commercial or financial relationships that could be construed as a potential conflict of interest.
